# Phase II study of capecitabine and cisplatin as first-line combination therapy in patients with gastric cancer recurrent after fluoropyrimidine-based adjuvant chemotherapy

**DOI:** 10.1038/sj.bjc.6602336

**Published:** 2005-01-18

**Authors:** H J Kang, H M Chang, T W Kim, M-H Ryu, H J Sohn, J H Yook, S T Oh, B S Kim, J-S Lee, Y-K Kang

**Affiliations:** 1 Division of Oncology, Department of Medicine, Asan Medical Center, 388-1 Pungnap-dong, Songpa-gu, Seoul 138-736, South Korea; 2 Department of Surgery, University of Ulsan College of Medicine, Asan Medical Center, Seoul, Korea

**Keywords:** capecitabine, cisplatin, first-line chemotherapy, advanced gastric cancer

## Abstract

To evaluate the efficacy and safety of capecitabine and cisplatin in patients with recurrent gastric cancer after fluoropyrimidine-based adjuvant therapy. Patients with histologically confirmed and measurable advanced gastric cancer that had relapsed after fluoropyrimidine-based adjuvant chemotherapy received oral capecitabine (1250 mg m^−2^ twice daily, days 1–14) and intravenous cisplatin (60 mg m^−2^ over 1 h, day 1) every 3 weeks. In total, 32 patients were enrolled, of whom 30 were evaluable for efficacy and 32 for safety. A median of 5 cycles (range 1–10) was administered. One patient achieved a complete response and eight had partial responses, giving an overall response rate of 28% (95% CI, 13–44%). The median time to progression and median overall survival were 5.8 months (95% CI, 4.1–7.5 months) and 11.2 months (95% CI, 5.5–16.9 months), respectively. Grade 3 neutropenia and thrombocytopenia were observed in 38 and 6% of patients, respectively. Grade 2/3 nonhaematological toxicities included diarrhoea (19%), stomatitis (19%) and hand-foot syndrome (31%). No grade 4 toxicity, neutropenic fever or treatment-related deaths occurred. Capecitabine in combination with cisplatin was effective and well tolerated as first-line treatment in patients with recurrent gastric cancer after fluoropyrimidine-based adjuvant chemotherapy.

Gastric cancer is the second most common cancer worldwide (Parkin *et al*, 1999). While the incidence of gastric cancer has been declining for several decades, it varies substantially between different racial and ethnic groups. For example, in South Korea, gastric cancer remains the most common and most fatal malignant neoplasm (Bae *et al*, 2002). Surgical resection is the only curative treatment currently available for gastric cancer, but the role of adjuvant chemotherapy after curative resection is unclear. A meta-analysis showed that adjuvant chemotherapy was associated with borderline statistically significant, but clinically insignificant, survival improvement (Earle and Maroun, 1999). A more recent meta-analysis (Janunger *et al*, 2002) of 21 randomised studies found a significant survival benefit for patients treated with postoperative adjuvant chemotherapy compared with controls (odds ratio (OR) 0.84, 95% confidence interval (CI) 0.74–0.96). However, when Western and Asian studies were analysed separately there was no benefit in the Western groups (OR 0.96, 95% CI 0.83–1.12). Recently, the North American Intergroup performed a prospective randomised study in 556 patients with advanced oesophagogastric cancer receiving postoperative adjuvant 5-FU/leucovorin plus radiotherapy (MacDonald *et al*, 2001). In this study, chemoradiation led to a significant improvement in median overall survival compared with surgery alone (36 *vs* 27 months, *P*=0.005). However, this study has been criticised in light of the poor overall survival, probably because inadequate surgery (D0 lymph node dissection) was performed in over 50% of the patients (Stahl, 2004). Overall, at the time of diagnosis, many patients have locally advanced unresectable or metastatic disease and, even after apparently complete resection, local and distant relapses are common. The prognosis of patients with recurrent gastric cancer is very poor, with the median duration of survival ranging from 3 to 5 months in untreated patients (Glimelius *et al*, 1997).

In patients with unresectable/metastatic disease, first-line chemotherapy is superior to best supportive care in terms of quality of life and overall survival (Murad *et al*, 1993; Pyrhonen *et al*, 1995; Glimelius *et al*, 1997). 5-FU is widely used for the treatment of gastric cancer and other gastrointestinal tumours. 5-FU in combination with cisplatin (FP regimen) is commonly used in advanced disease because of the activity of both drugs when administered as single agents. In randomised phase III trials in advanced gastric cancer, FP led to improved response rates compared with 5-FU, doxorubicin and mitomycin (FAM) or 5-FU single-agent therapy (Kim *et al*, 1993), and showed a trend towards improved response rates compared with 5-FU, doxorubicin and methotrexate (FAMTX) or etoposide, leucovorin and bolus 5-FU (ELF) (Vanhoefer *et al*, 2000). However, few studies have evaluated the efficacy of first-line treatment in patients with gastric cancer that has relapsed after fluoropyrimidine-based adjuvant chemotherapy. Furthermore, first-line chemotherapy after relapse is often associated with pronounced adverse effects and response rates rarely exceed 20% (Hill and Cunningham 1998). For these reasons, novel compounds with activity and less toxicity are needed in this setting.

The oral fluoropyrimidine capecitabine (Xeloda®) was designed to generate 5-FU preferentially in tumour tissue and to mimic a continuous infusion of 5-FU while minimising systemic 5-FU exposure. Following absorption, capecitabine is metabolised in a three-step metabolic process, the final step being conversion to 5-FU by thymidine phosphorylase (TP): tumour selectivity results from the significantly greater TP activity in tumour tissue compared with healthy tissue (Miwa *et al*, 1998; Schüller *et al*, 2000). In preclinical xenograft models, oral capecitabine has been shown to be highly active against gastric cancer (Ishikawa *et al*, 1998; Ishitsuka, 2000). This finding was subsequently extended to the clinic in a phase II study of previously untreated patients with advanced gastric cancer, in which capecitabine 1250 mg m^−2^ twice daily on days 1–14 every 3 weeks was both active (overall response rate 28%; stable disease 36%) and well tolerated (Hong *et al*, 2004). In addition, capecitabine showed activity in a preclinical xenograft model of a 5-FU-resistant tumour (Cao *et al*, 1997) and some activity in patients with metastatic colorectal cancer refractory to 5-FU/leucovorin chemotherapy (Lee *et al*, 2003).

As oral capecitabine is a highly active single agent and its safety profile differs from that of cisplatin with little overlap of key toxicities, capecitabine combined with cisplatin is an appealing and convenient alternative to 5-FU/cisplatin. In a recent phase II study (Kim *et al*, 2002), capecitabine plus cisplatin was active and well tolerated as first-line chemotherapy. In the present phase II study we evaluated the efficacy and safety of the same combination regimen as first-line treatment in patients with gastric cancer recurring after fluoropyrimidine-based adjuvant chemotherapy.

## MATERIALS AND METHODS

### Patient selection

Patients were considered eligible if they had histologically confirmed advanced gastric cancer with at least one measurable target lesion according to RECIST guidelines (Therasse *et al*, 2000) (diameter of ⩾2 cm) that had relapsed after previous fluoropyrimidine-based adjuvant chemotherapy. In addition, patients had to be 18–75 years old, have an Eastern Cooperative Oncology Group (ECOG) performance status of 0–2, have adequate liver, kidney, and bone marrow function, and to have received no prior treatment with capecitabine or platinum compounds. Patients with unresolved bowel obstruction or malabsorption syndrome were excluded. The protocol was approved by the institutional review board of the Asan Medical Center, and all patients gave written informed consent before enrolment.

### Treatment schedule

Capecitabine was administered orally at a dose of 1250 mg m^−2^ twice daily according to the standard intermittent schedule (14 days of treatment followed by a 7-day rest period, every 3 weeks). Cisplatin was administered intravenously on day 1 (before the first dose of capecitabine) at a dose of 60 mg m^−2^ for 1 h with hydration, and repeated every 3 weeks. The hydration procedure consisted of 1 l of normal saline (containing 20 mEq of KCl and 8 mEq of MgSO_4_) infused intravenously for 2.5 h both before and after cisplatin infusion, giving a total of 2 l of saline infused over a 5-h period. In addition, intravenous furosemide 20 mg was given 30 min before infusing cisplatin. Mannitol was not used. A serotonin antagonist and dexamethasone were routinely given before cisplatin administration to prevent emesis. Treatment was given in the outpatient clinic and continued until disease progression, unacceptable adverse events, or withdrawal by the patient.

### Evaluation of safety and dose modification

Safety was evaluated before each treatment cycle according to the National Cancer Institute Common Toxicity Criteria (NCI-CTC), version 2.0. To begin the next treatment cycle, each patient was required to have a platelet count ⩾75 × 10^9^ l^−1^, a neutrophil count ⩾1 × 10^9^ l^−1^ and resolution or improvement of clinically significant nonhaematological adverse events (excluding alopecia) to grade 1 or 0. Dose adjustments (interruption and/or reduction) and discontinuation in response to adverse events were made for each drug according to previous guidelines and depended on classification, grade and frequency of occurrence (Kim *et al*, 2002). Dose adjustment criteria for cisplatin were based on serum creatinine levels immediately prior to each cycle: if serum creatinine was <1.5 mg dl^−1^, full-dose cisplatin was given; if serum creatinine was 1.5–2.5 mg dl^−1^, the cisplatin dose was reduced by 50%; if serum creatinine was >2.5 mg dl^−1^, the patient was excluded from the study. Patients were withdrawn from study treatment (but still followed-up) if treatment was delayed for more than 2 weeks.

### Assessment of compliance and dose intensity

Compliance with capecitabine treatment was monitored by questioning patients and counting their remaining pills at each outpatient visit. The ratio of the actual administered dose to the scheduled dose was then calculated. Dose intensity was defined as the total amount of drug given (mg m^−2^) divided by the number of weeks.

### Pretreatment, follow-up studies and response evaluation

Physical examination and chest X-rays were carried out before each chemotherapy cycle, and complete blood counts and biochemical tests were performed before and on day 15 of each cycle. Response evaluation was performed by computed tomography (CT) scan every 2–3 cycles until the tumour progressed. Tumour response was classified on the basis of the response evaluation criteria defined by RECIST guidelines (Therasse *et al*, 2000), and responses were required to last longer than 4 weeks.

### Statistical analysis

All enrolled patients were included in the intention-to-treat (ITT) analysis of efficacy. The trial was conducted according to the two-stage Gehan design (Simon 1987) with response rate as the primary end point. We planned to enrol at least 25 evaluable patients, with a target minimum response rate of 20%. If no objective response was seen among the first 14 patients in the study, the probability of a response rate ⩾20% would be <5%, and the study was to be discontinued. One or more responses would indicate that continuation was warranted, and at least 25 patients would be required to estimate a response rate with a standard error of approximately 10%. The number of patients enrolled was increased to 32 patients to better estimate the response rate. The Fisher's exact test was used to compare late *vs* early relapse groups and the different adjuvant regimens.

Time to progression (TTP), survival and duration of response were secondary end points and were estimated using the Kaplan–Meier method. The duration of response was defined as the interval from the onset of complete response (CR) or partial response (PR) until first evidence of disease progression. If death occurred before progression was documented, the date of death was assumed to be the date of progression. TTP was calculated from the date of entry into the study until the date of progression, and overall survival was measured from the date of entry to the date of last follow-up or death.

## RESULTS

### Patient characteristics

A total of 32 patients were enrolled between October 2000 and April 2003. Baseline characteristics, which are shown in Table 1, show a relatively standard gastric cancer population (with more males than females).

### Efficacy and survival

A total of 30 patients were evaluable for response (Table 2). One patient was not evaluable because of loss to follow-up after the first cycle of treatment, and the second patient withdrew consent. One CR and eight PRs were observed, giving an overall response rate of 28% (95% confidence intervals (CI), 13–44%) in the ITT analysis (Table 2).

There was a numerically superior overall response rate in patients whose tumour relapsed more than 6 months (late relapse group) compared with those whose tumour relapsed within 6 months of completing adjuvant chemotherapy (early relapse group) (39 *vs* 21%), although the difference did not reach statistical significance (*P*=0.427; Table 2). There was also no significant difference in overall response rate in patients who received doxifluridine±mitomycin-C (40%) compared with 5-FU+doxorubicin+mitomycin-C (23%, *P*=0.407; Table 2).

**Figure 1:**
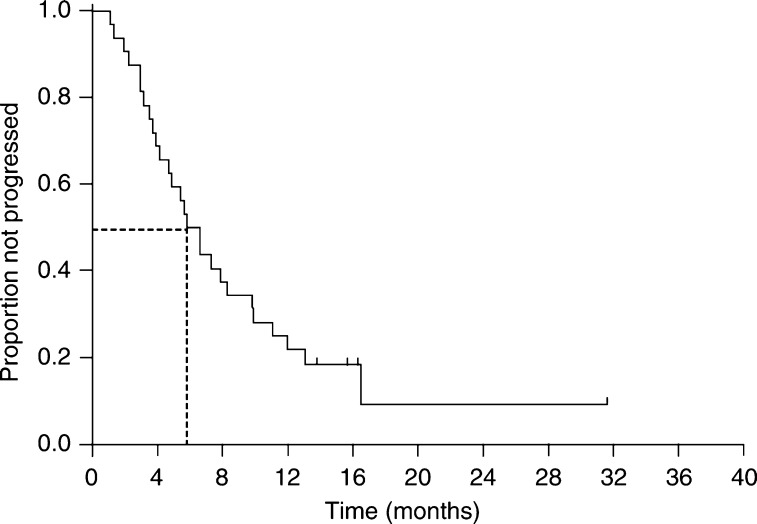
Time to disease progression for all patients.

**Figure 2:**
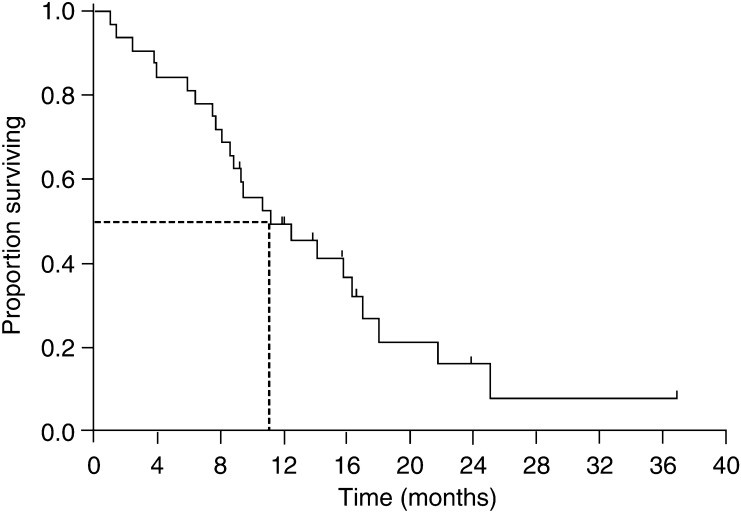
Overall survival for all patients.

The median duration of response in the nine responding patients was 8.5 months (range 3.6–29.6 months). The median follow-up period was 19.4 months (range 9.2–39.8 months). The median TTP for all patients was 5.8 months (95% CI, 4.1–7.5 months; Figure 1). The median overall survival was 11.2 months (95% CI, 5.5–16.9 months; Figure 2), with a 1-year survival rate of 49% (95% CI, 32–66%). Although there was a trend towards a more prolonged overall survival (14.1 *vs* 9.3 months, *P*=0.075) and TTP (8.3 *vs* 5.4 months, *P*=0.072) in the late relapse group compared with the early relapse group, the differences did not reach statistical significance.

### Adverse events

A total of 173 treatment cycles (median 5; range 1–10 cycles) were administered, of which there are no data for one cycle because one patient was lost to follow-up. The frequencies of treatment-related haematological and nonhaematological adverse events are shown in Table 3. The most common treatment-related haematological adverse event was neutropenia, which occurred at grade 3 intensity in 12 patients (38%). No patient experienced grade 4 neutropenia or febrile neutropenia. Grade 2 hand-foot syndrome was also relatively common, occurring in 10 patients (31%). There were no treatment-related deaths.

Treatment interruption or dose reduction was required in 71 cycles. In total, 20 patients (62.5%) required dose reductions, which were due to haematological adverse events (12 of 20 patients; 60%), hand-foot syndrome (three patients; 15%), nausea/vomiting (two patients; 10%), stomatitis (one patient; 5%), and diarrhoea (one patient; 5%). Treatment was delayed in 16 patients (50%) as a result of haematological adverse events (13 patients; 81.3%), hand-foot syndrome (two patients; 12.5%), nausea/vomiting (one patient; 6.3%) and stomatitis (one patient; 6.3%). There was no treatment interruption or dose reduction with cisplatin.

The median dose intensity for all treatment cycles was 8902 mg m^−2^ week^−1^ (range 4298–15576 mg m^−2^ week^−1^) for capecitabine and 19.2 mg m^−2^ week^−1^ (range 15.0–27.5 mg m^−2^ week^−1^) for cisplatin, corresponding to 76 and 96%, respectively, of the planned dose intensities. Although patient compliance with taking their prescribed number of capecitabine tablets was good (97% during the first six cycles), the dose intensity of capecitabine decreased progressively during the first six cycles and fell below 80% of that planned after the second treatment cycle. Conversely, the dose intensity of cisplatin was well maintained throughout the first 6 cycles.

## DISCUSSION

The present study suggests that the combination of capecitabine and cisplatin is an effective and well-tolerated regimen for the first-line treatment of patients with gastric cancer recurrent after fluoropyrimidine-based adjuvant chemotherapy. This combination regimen demonstrated promising efficacy, with a tumour response rate of 28%, a median TTP of 5.8 months and a median overall survival of 11.2 months. The efficacy demonstrated in the present study may be a function of additive or synergistic antitumour activity between the two agents also observed in other studies in a variety of gastrointestinal cancers (Evans *et al*, 2002; Kim *et al*, 2002, 2003; Pivot *et al*, 2003). The lack of prior exposure to cisplatin may have also played a role in the response to the capecitabine–cisplatin combination.

It is difficult to compare the results of the present study with other studies, as there is little information in the literature on response to first-line chemotherapy after relapse of advanced gastric cancer following fluoropyrimidine-based adjuvant chemotherapy. The results of this study seem to be similar or somewhat superior to those achieved with other regimens used in this setting, such as weekly high-dose infusional 5-FU/leucovorin, docetaxel or irinotecan (Futatsuki *et al*, 1994; Vanhoefer *et al*, 1994; Graziano *et al*, 2000; Giuliani *et al*, 2003). Weekly high-dose infusional 5-FU/leucovorin has been reported to achieve a response rate of 18% and a median overall survival of 5 months (Vanhoefer *et al*, 1994), whereas docetaxel or irinotecan showed objective response rates in the range of 5–27% and overall survival times ranging from 3.5 to 10.2 months (Futatsuki *et al*, 1994; Graziano *et al*, 2000; Giuliani *et al*, 2003). However, since our study population was limited to patients with gastric cancer recurrent after previous adjuvant chemotherapy, and excluded those with metastatic advanced gastric cancer who failed first-line chemotherapy, the results of this study should be interpreted cautiously. Nevertheless, considering that single-agent cisplatin is associated with a response rate of approximately 19% when used as first-line therapy (Aabo *et al*, 1985; Perry *et al*, 1986), we would not have expected to observe a response rate much greater than this by combining a fluoropyrimidine (i.e. capecitabine) with cisplatin in patients who had recurrent disease after prior fluoropyrimidine-based adjuvant chemotherapy. Therefore, the current response rate of 28% is encouraging.

The results of the present study are similar to our previous study of the same regimen as a first-line treatment in previously untreated patients with advanced gastric cancer (Kim *et al*, 2002) with regard to both median overall survival (11.2 *vs* 10.1 months) and median TTP (5.8 *vs* 6.3 months), although the objective response rate was lower (28 *vs* 55%). It is logical that the same chemotherapeutic regimen should have a lower response rate in patients who have previously received fluoropyrimidine-based adjuvant therapy than those who have not. The similar TTP and overall survival may have been a result of differences in tumour burden between the two study populations at the start of chemotherapy; in the current study, 34% of patients had involvement of more than one organ compared with 46% in our previous study. In addition, these similar survival results may be related to the second-line treatment received by patients; 47% of patients received second-line treatment in the present study compared with 29% of patients in our previous study. Also in the current study, patients with early relapse, who may have developed fluoropyrimidine-resistant tumours, showed a favourable response rate, TTP and overall survival, suggesting that the combination of capecitabine and cisplatin may overcome drug resistance to fluoropyrimidines. However, further studies are required to verify this observation.

Owing to the limited response duration, TTP and overall survival in patients with gastric cancer, safety and tolerability are important considerations in the assessment of new treatment regimens. The combination of capecitabine and cisplatin has previously shown good antitumour efficacy with a favourable safety profile as first-line chemotherapy in advanced gastric (Kim *et al*, 2002) and biliary cancer (Kim *et al*, 2003), and also as salvage treatment in previously treated head and neck cancer patients (Pivot *et al*, 2003). In the present study, adverse events were generally mild and manageable without the need for hospitalisation. There were no treatment-related deaths or cases of febrile neutropenia, despite 38% of patients developing grade 3 neutropenia. Hand-foot syndrome was common, but severe cases were successfully prevented through strict adherence to a predefined dose modification schedule. These data suggest that capecitabine in combination with cisplatin can be administered safely in an outpatient clinic setting.

Compliance with capecitabine, which is very important for oral chemotherapeutic agents, was generally very good. However, the median dose intensity for capecitabine was 76% of that planned because of dose reductions or delays primarily associated with neutropenia. In addition, the dose intensity of capecitabine gradually decreased over the first six treatment cycles, just as in other phase II studies of capecitabine plus cisplatin as first-line chemotherapy (Kim *et al*, 2002, 2003). Therefore, we recommend that the starting dose of capecitabine be reduced to 1000 mg m^−2^ twice daily on days 1–14 every 3 weeks in any future evaluations of this combination.

It is interesting to note that triple-drug combinations, such as ECF (Webb *et al*, 1997), have shown particularly favourable responses in the first-line treatment of previously untreated advanced gastric cancer. Recently, we conducted a phase I/II study of a new triple-drug combination of docetaxel–capecitabine–cisplatin as first-line therapy in advanced gastric cancer and observed very promising efficacy and acceptable safety (Kang *et al*, 2004). Capecitabine/cisplatin in combination with epirubicin (ECX) is also being evaluated in a randomised phase III study (REAL2 trial) as first-line therapy for previously untreated advanced gastro-oesophageal cancer (Sumpter *et al*, 2003); another triple combination including capecitabine (epirubicin/oxaliplatin/capecitabine: EOX) is being examined in this trial together with the complimentary regimens containing 5-FU (ECF and epirubicin/oxaliplatin/5-FU: EOF). An international phase III trial is also underway to evaluate replacing infusional 5-FU with capecitabine in 5-FU/cisplatin combination chemotherapy in first-line advanced gastric cancer.

In conclusion, the combination of capecitabine and cisplatin is effective and well tolerated as a first-line treatment for gastric cancer recurrent after prior fluoropyrimidine-based adjuvant chemotherapy. It would be interesting to speculate that addition of a third agent (e.g. epirubicin, docetaxel, paclitaxel, irinotecan) to capecitabine/cisplatin might improve this response in recurrent gastric cancer after prior fluoropyrimidine-based adjuvant chemotherapy. With the increasing adoption of adjuvant chemotherapy and chemoradiotherapy after surgery for advanced gastric cancer, there will be an expansion of studies into the use of first-line chemotherapy regimens for recurrence of gastric cancer following prior adjuvant therapy.

## Figures and Tables

**Table 1:** Patient characteristics

**Characteristic**	**No.**	**%**
*Age (years)*		
Median	60	
Range	38–73	

*Sex*		
Male	22	69
Female	10	31

*ECOG performance status*		
0	1	3
1	30	94
2	1	3

*Time between end of adjuvant chemotherapy and relapse*		
⩽6 months	19	59
>6 months	13	41

*Prior adjuvant chemotherapy*		
5-FU+doxorubicin+mitomycin-C	22	69
Doxifluridine±mitomycin-C	10	31

*Metastatic sites*		
Liver	15	47
Abdominal lymph node	13	41
Peritoneum	6	14
Cervical lymph node	4	13
		
*No. of metastases*		
1	21	66
⩾2	11	34

**Table 2:** Antitumour efficacy

			**Time from end of adjuvant therapy to relapse**				**Prior adjuvant therapy**			
	**All patients^a^ (*n*=32)**		**>6 months (*n*=13)**		**⩽6 months (*n*=19)**		**5-FU+doxorubicin+ mitomycin-C (*n*=22)**		**Doxifluridine± mitomycin-C (*n*=10)**	
	**No.**	**%**	**No.**	**%**	**No.**	**%**	**No.**	**%**	**No.**	**%**
Complete response	1	3	1	8	0	0	0	0	1	10
Partial response	8	25	4	31	4	21	5	23	3	30
Stable disease	17	53	7	54	10	53	13	59	4	40
Progressive disease	4	13	1	8	3	16	2	9	2	20
Not evaluable	2	6	0	0	2	11	2	9	0	0

a Intention-to-treat analysis.

**Table 3:** Most common treatment-related adverse events (>10% of patients)

	**Grade (% of patients)^a^**				
	**1**	**2**	**3**	**4**	**All grades**
Anaemia	31	50	16	0	97
Neutropenia	13	38	38	0	89
Hand-foot syndrome	50	31	0	0	81
Asthenia	34	44	0	0	78
Leukopenia	28	41	9	0	78
Nausea	28	47	0	0	75
Neuropathy	47	19	0	0	66
Thrombocytopenia	41	13	6	0	60
Diarrhoea	38	19	0	0	57
Constipation	19	31	0	0	50
Vomiting	19	28	0	0	47
Stomatitis	28	16	3	0	47
Hyperbilirubinaemia	13	6	0	0	19
Elevated transaminases	13	0	0	0	13

a NCI-CTC, version 2.0.
